# A review of migratory *Alosidae* marine ecology in the northwest Atlantic

**DOI:** 10.1111/jfb.15977

**Published:** 2024-11-10

**Authors:** Caliyena R. Brown, Ava J. A. Sergio, Caitlin S. Bate, Natalie Koopman, Joshua B. Roland, Oscar D. P. Notman‐Grobler, Paris M. B. Mastrodimitropoulos, Morgan L. Piczak, Robert J. Lennox

**Affiliations:** ^1^ Department of Biology Dalhousie University Halifax Nova Scotia Canada; ^2^ Ocean Tracking Network Dalhousie University Halifax Nova Scotia Canada

**Keywords:** anadromous, conservation, fisheries management, forage fish, migration, movement ecology

## Abstract

Migratory animals play a crucial role in connecting distinct habitats by transferring matter and energy across ecosystem boundaries. In the North Atlantic, anadromous species exemplify this through their movement between freshwater and marine environments. Alosids, including species such as alewife (*Alosa pseudoharengus*), blueback herring (*Alosa aestivalis*), and American shad (*Alosa sapidissima*), exhibit this migratory behavior to maximize growth and fecundity and are, therefore, vital components of Atlantic coastal ecosystems. Despite their ecological importance, these species have experienced considerable population declines. Due to a research focus on dams and the freshwater phase of their ecology, the marine ecology of Alosids remains much less understood, potentially hindering effective management. This paper synthesizes current knowledge on the marine ecology of anadromous alewife, blueback herring, and American shad in the northwest Atlantic, focusing on life‐history aspects, migratory patterns, and foraging behavior at sea. The paper also outlines current fisheries management and the anthropogenic threats these species face during their marine phase. We identified knowledge gaps regarding marine distribution, migration routes, impacts of climate change on movement and behavior, population dynamics, and the identification of gaspereau. By identifying gaps in the literature, we highlight research needs, emphasizing the role of telemetry studies in tracking marine movements and the impact of climate change on habitat use. Addressing these gaps through targeted research on marine ecology and movement patterns is essential for developing informed management strategies aimed at increasing Alosid populations.

## 1 INTRODUCTION

Migration moves matter rapidly across relatively impenetrable boundaries (Cooke et al., 2024). Migratory animals are, therefore, important conduits of matter and energy at a landscape scale and connect distinct ecosystems and habitats (Bauer & Hoye, 2014; Post & Walters, 2011). In coastal ecosystems, movement between fresh water and the ocean allows fish species to maximize growth and fecundity, thereby, enhancing their lifetime fitness (e.g., Atlantic salmon, *Salmo salar*; Thorstad et al., 2011). In the temperate North Atlantic, several species exhibit anadromy, spawning in the relative safety of the oligotrophic fresh waters while exploiting the seasonal productivity of the marine environment (Samways et al., 2018). The ecology of freshwater spawning migrations of the fishes within the North Atlantic has received extensive research attention, although much less is known about oceanic movements, which could be hindering the effective management of these species. Further, salmonids are represented in the literature far more relative to other diadromous species, including Alosids, which have many paucities regarding their marine ecology.

**FIGURE 1:**
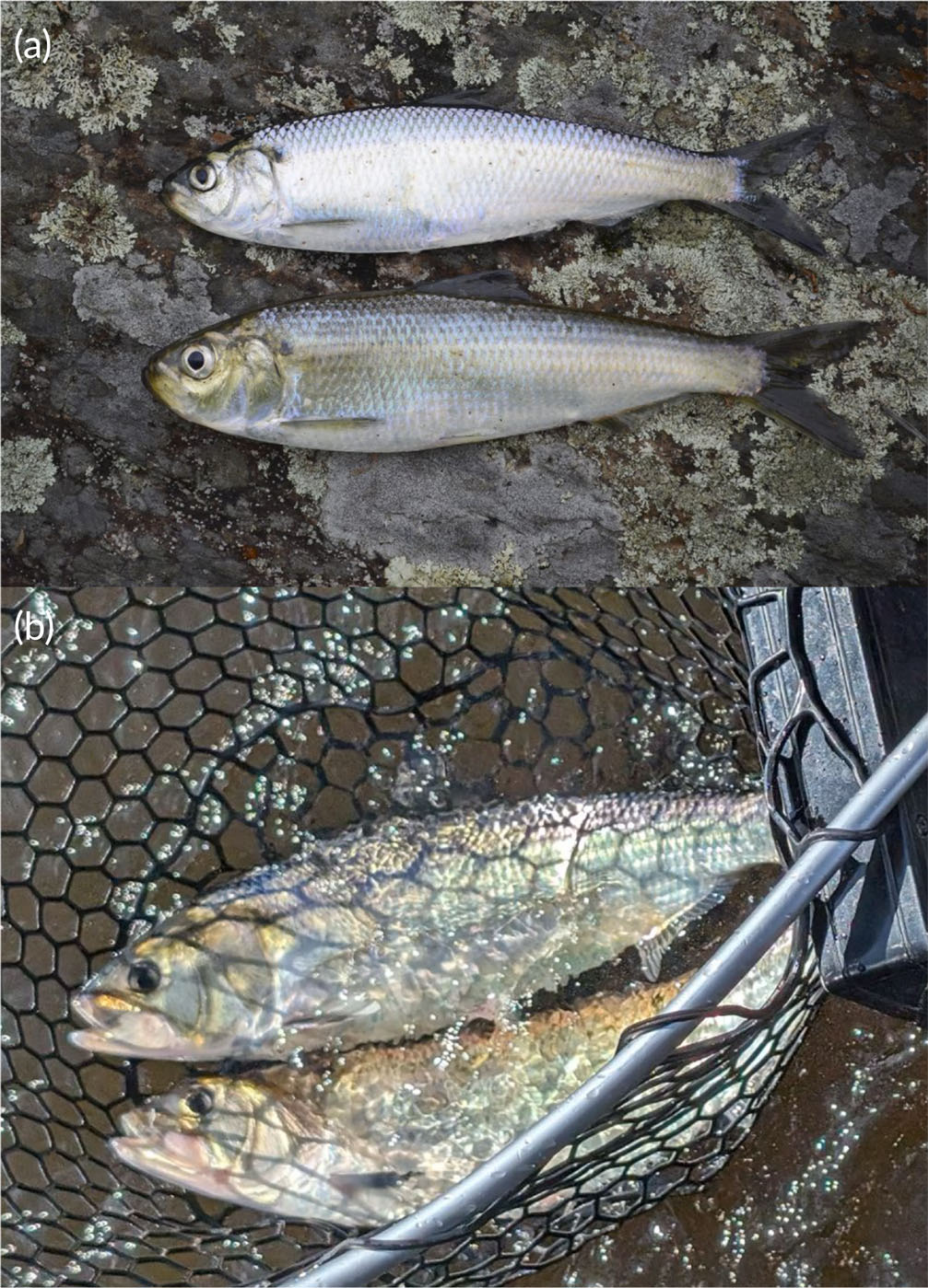
(a) Alewife and blueback herring (photo credit: Christopher Bartlett) and (b) American shad (photo credit: Mikey Lennox).

Among the most productive and abundant migratory diadromous species are the Alosids, which are an anadromous group of fishes that include six species in North America, including alewife (*Alosa pseudoharengus*) and blueback herring (Figure 1a; *Alosa aestivalis*; collectively referred to as “gaspereau” or “river herring” because they are virtually indistinguishable to the eye, herein gaspereau), skipjack shad (*Alosa chrysochloris*), hickory shad (*Alosa mediocris*), Alabama shad (*Alosa alabamae*), and American shad (*Alosa sapidissima*; Figure 1b; Bi et al., 2021). Considered to be forage species, Alosids provide a critical link between the lower food web and higher trophic predators across diverse taxa of fish, marine mammals, and birds (Schmitt et al., 2017), highlighting their ecological importance in the marine food web. Additionally, Alosids are highly productive across the Atlantic coast and have supported substantial commercial and recreational fisheries in Canada and the United States (McPhee, 2003). However, Alosids have experienced considerable population declines attributable to several anthropogenic stressors such as dams (Hasselman & Limburg, 2012; Mather et al., 2012). For instance, yearly landings of American shad from several rivers in Maine were estimated to reach 2 million fish prior to damming in the 1930s (Saunders et al., 2006). More recently, annual returns of American shad were estimated at ≤2000 fish (Saunders et al., 2006). Likewise, landings of gaspereau off the Mid‐Atlantic coast exceeded 25,000 t until the 1970s, but since 1993, they have declined to less than 1000 t (Kocik, Haas‐castro, et al., 2009).

Alosids play a critical role in coastal ecosystems, and although fresh water is relied on for spawning and rearing, most of the life of the anadromous Alosids is at sea (Turner & Limburg, 2016), where they are relatively challenging to study. In the ocean, Alosids are difficult to observe, and current landing records are limited; therefore, many questions about their marine ecology remain unanswered. It is necessary to fill these knowledge gaps to enable more effective management of these species and to manage marine ecosystems for resilience. The objective of this paper is to synthesize the current knowledge about the marine ecology of the anadromous Alosids native to Canadian waters: the alewife, blueback herring, and American shad. For each of these three Alosids, we examine several aspects of their life history, including diet, growth, spawning, and death. Next, we form a comprehensive summary of their movement ecology, including both spawning migrations and foraging behavior at sea. From there, we identify threats and current fisheries management. Lastly, we highlight key knowledge gaps and directions for future studies that could help address these paucities and aid in supporting population recovery of the forage fishes that form the base of the food web.

## 2 ALEWIFE

### 2.1 Life history

#### 2.1.1 Diet

Alewife begin their lives in freshwater, feeding on small, planktonic crustacean nauplii before they grow and move toward the sea (Bozeman Jr & Van Den Ayvle, 1989). Although the pelagic diet of invasive alewife in the Laurentian Great Lakes is well understood (e.g., Pothoven & Vanderploeg, 2004; Stewart et al., 2009; Strus & Hurley, 1992), thorough descriptions of the diet of anadromous alewife in the Atlantic are still limited. Some insights into their diet in the marine environment were, however, gathered from stomach content analyses from alewife caught along the continental shelf from New England to Cape Hatteras, North Carolina (Bowman et al., 2000). In that study, non‐decapod crustaceans represented the majority of the alewife's prey by weight, and adults were found to primarily filter‐feed on copepods and krill. However, the stomach contents of larger individuals (>20 cm) included mollusks, cnidarians, and juvenile fish, suggesting an ontogenic shift in alewife from primarily filter feeding to a mixed strategy that includes predation on a higher diversity of taxa (Table 1; Bowman et al., 2000).

**TABLE 1:** Summary of life‐history aspects and behavior in the marine environment for each alewife (*Alosa pseudoharengus*), blueback (*Alosa aestivalis*), and American shad (*Alosa sapidissima*).

Aspect		Alewife	Blueback herring	American shad
Life history	Diet	Mainly prey on non‐decapod crustaceans Adults also feed on copepods and krill Larger individuals (>20 cm) feed on mollusks, cnidarians, and bony fish	Consume both pelagic and benthic prey, but more selective than alewife Juveniles primarily feed on zooplankton Adults also consume zooplankton, as well as small fishes, fish eggs, and small crustaceans	Adults cannibalize juveniles in nearshore areas on their migration back to sea At sea, adults feed on copepods, mysids, and small fish
	Growth	Evidence of 114–127 mm of growth in their first year in the Chesapeake Bay estuary Adults are sexually dimorphic; females spend longer at sea and reach a larger average length of 284.3 mm, whereas males average 271.6 mm Maturation at sea takes 3–6 years Lower latitude populations are shorter lived and quicker to mature VBGF growth parameters: L∞ = 291.67 mm (males) – 310.48 mm (females) K = 0.4 (females) – 0.441 (males) t_0_ = 0.103 (females) – 0.142 (males)	Adults grow up to 197–322 mm in fork length Females are larger than males; 289 mm and 277 mm, respectively Growth rates plateau after spawning, and there is little growth between spawning events Energetic trade‐off between frequent spawning and at‐sea growth (multiple spawners are smaller) Maturation at sea takes 4–5 years for females and 3–4 years for males VBGF growth parameters: L∞ = 231.33 mm (males) – 259.85 mm (females) K = 0.469 (females) – 0.590 (males) t_0_ = 0.283 (females) – 0.338 (0.338)	Juveniles migrate to sea when they reach 38–114 mm Shad are larger than alewife and blueback and reach up to 760 mm Females are larger than males Northern populations are larger than southern contingents Maturation at sea takes 2–6 years; males typically mature in 2 years, females mature in 3–4 years Average VBGF growth parameters: L∞ = 481 mm K = 0.44 t_0_ = −0.32
	Spawning	Adults exhibit spawning site fidelity and return to their natal stream or pond using olfactory cues Higher‐latitude populations demonstrate more frequent iteroparity Interannual returns are higher among males Precede blueblack in spawning migrations of shared rivers	Adults return to their natal river to spawn Exhibit iteroparity and will spawn up to four times Gravimetric fecundity positively correlates with age and decreases with increasing latitude Spawning season lengthens with increasing latitude Spawning can take place in brackish water Return to spawn later in the spring than alewife	Adults exhibit spawning site fidelity and return to their natal river to spawn; low levels (3%) of straying have been observed Northern populations are iteroparous and can spawn up to five to seven times, whereas the southern populations are semelparous Semelparity is observed only in St. Johns River, Florida, and Ogeechee River, Georgia
	Mortality	Lower‐latitude populations live 3–4 years; northern counterparts live up to 9–10 years Predation is a primary cause of mortality Energy movement and nutrient input in the marine environment are understudied but likely comparable to the contributions in fresh water Key driver of predator movement (i.e., Atlantic cod and other gadids)	Maximum age of an individual observed in sampling studies were 8 and 12 years old Females live longer than males Males are more abundant in younger age classes Predation by fish, sea birds, and marine mammals is a leading cause of mortality in the marine environment	Can live up to 13 years Natural mortality rates differ based on latitude due to reproductive styles; northern populations live longer Predation by sharks, several fish species, and marine mammals significantly contribute to adult mortality Adults commonly die during spawning migrations
Behavior	Migration	Range from Carolina to Newfoundland Juveniles join large schools of similar‐sized individuals after they leave their spawning river Schooling with menhaden or blueback is common Move northward and inshore in the spring and offshore and southward in the fall Found along the Mid‐Atlantic coastline in the winter and spring Movement is regulated by zooplankton productivity and by abiotic factors, such as temperature, tidal currents, salinity, and depth Marine temperatures > 14°C are avoided Water temperatures 5–10°C cue spawning migrations Spawning begins late March in the south and progressively later into July further north	Marine range from Florida to Nova Scotia Some migratory contingents remain resident in estuaries near spawning rivers; some undertake offshore migrations Summer aggregations consist of a mixed stock of contingents from multiple rivers Move southward in the fall to aggregate offshore in deeper waters in overwintering sites in Mid‐Atlantic Bight and along Scotian Shelf Migration follows temperature contours in northern limit of range and zooplankton availability Move to mid‐depth, coastal waters in the spring Migration to spawning rivers when water temperatures range 14–22°C Spawning begins later than blueback herring, in late April	Migrate from Florida to Newfoundland Intraspecific schools of juveniles and postspawning adults overwinter in deep waters offshore Florida, the Mid‐Atlantic Bight, and along the Scotian Shelf Overwintering aggregations are heterogenous mixtures of populations from many rivers Spawning migrations occur when water temperatures are between 8 and 26°C Southern populations begin northward spawning migrations in January, and northern populations begin spawning migrations progressively later into the spring as latitude increases
	Foraging	Use a mixture of inshore and offshore foraging areas Feeding schools are mixed stock of several populations Have diel vertical migrations following zooplankton Use both particulate (during day) and filter‐feeding (during night) strategies Zooplankton productivity and foraging partially regulate movement at sea Forage in nearshore habitat until water is too warm (>14°C)	School as single‐species aggregations or with alewife to optimize feeding Undertake diel vertical migrations following zooplankton throughout water column Demonstrate active foraging and filter‐feeding Feeding migrations from North Carolina to Nova Scotia have been recorded Some populations utilize inshore embayments for feeding in the summer	Juveniles join large intraspecific feeding schools along the coast Demonstrate diel feeding patterns following zooplankton Primarily feed in the evening Summer foraging grounds include surface waters in the inner Bay of Fundy, the inner Gulf of St. Lawrence, and off Newfoundland and Labrador Majority of foraging occurs in the marine environment Adults demonstrate passive filter feeding and active foraging

Abbreviation: VBGF, von Bertalanffy growth function.

#### 2.1.2 Growth

In the Chesapeake Bay estuary, juveniles were found to grow to about 114–127 mm long in their first year (Table 1; Hildebrand & Schroeder, 1927); however, once alewife enter the sea, there is a distinct lack of information regarding their rate of growth from the time they enter the ocean to when they return inshore. Several studies have shown that alewife are sexually dimorphic where females spend a longer time at sea and reach a larger average adult length of 284.3 mm (standard deviation = 15.77), whereas males reach an average length of 271.6 mm (standard deviation = 13.09; Table 1; Fay et al., 1983; Marjadi et al., 2019). The observed sexual dimorphism is also exhibited in the model parameters from the von Bertalanffy growth function (VBGF; Bertalannfy, von L., 1938) where L∞ is the mean asymptotic length of fish, K is the Brody growth coefficient and represents how quickly fish approach L∞, and t_0_ is the hypothetical age at which fish length is equal to zero (*x*‐intercept; Gilligan‐Lunda et al., 2021). For alewife, L∞ ranged from 291.67‐ (males) to 310.48 mm (females), K ranged from 0.4 (females) to 0.441 (males), and t_0_ ranged from 0.103 (females) to 0.142 (males; Messieh, 1977). Alewife take 3–6 years to mature at sea before returning to rivers to spawn (Table 1; Loesch, 1987). Individuals at lower latitudes are typically shorter lived and quick to mature, which is consistent with the latitudinal relationship across ectotherms (Table 1; Munch & Salinas, 2009).

#### 2.1.3 Spawning

Alewife preferentially return to their natal stream or pond, relying on olfactory mechanisms to detect the odor of the water from which they hatched (Table 1; Thunberg, 1971). The frequency of iteroparity in alewife populations is latitudinally influenced, with northern latitudes having a higher percentage of repeat spawners annually (Table 1; Fay et al., 1983). Iteroparity also varies at smaller spatial scales between sites, suggesting that river‐specific drivers, like anthropogenic impacts (habitat degradation, overfishing, dams, and loss of river connectivity), are primary determinants of the survival and fidelity of anadromous alewife populations (Hare et al., 2021; Spares et al., 2023). In the Gaspereau River in Nova Scotia, the alewife stock is heavily impacted by overfishing; of the previously spawned adults, only 11.1% of males and 7.4% of females returned the following years, compared to an estimated 50% return rate in rivers with minimal anthropogenic stressors (Gibson, 2000). Unlike salmonids, for which males expend more energy spawning and females have higher rates of repeat spawning (Jonsson et al., 1991), interannual returns are higher among male alewife than females (Table 1). However, this may be attributed to artificial selection, with the longer mean length of females at spawning age increasing their likelihood of being caught in gillnets, decreasing their survival during spawning season (Spares et al., 2023).

#### 2.1.4 Mortality

Southerly alewife live only 3–4 years, compared to their northern counterparts, which can live up to 9–10 years (Table 1; Fay et al., 1983). Predation is a primary cause of natural mortality, and alewife contribute to the diet of piscivorous fishes, birds, and mammals (Hare et al., 2021). Freshwater predation is a major bottleneck, and some predators may feed exclusively on alewife during their spawning migration (Hare et al., 2021), whereas predation at sea is more diffuse. Anadromous alewife store substantial energy from the ocean in their fatty tissues, contributing to the nutrient input of freshwater systems following their annual spawning events. Similar subsidies to the marine environment must be relevant, although they are less commonly considered than the subsidies provided to freshwater systems for anadromous species (Table 1; Dias et al., 2019). Although there is ample research on marine isotopes carried by anadromous fishes into fresh water (Durbin et al., 1979), there is little comparable work to find freshwater isotopic signatures in the marine environment that might suggest how alewife provide a reciprocal subsidy to the ocean via their anadromy. Nevertheless, alewife have shown to be significant to the diets of demersal groundfish, indicating a high degree of predation mortality at sea (Link & Garrison, 2002). Young of year (YOY) alewife immigrating from estuaries to the northeastern coastal shelf have even been suggested to influence the movement behavior of predator species (i.e., co‐migration; Hare et al., 2021). Historically, the pursuit of alewife by Atlantic cod (*Gadus morhua* and other gadids) may have been a key driver of gadid movement into estuaries and river mouths where alewife aggregate to spawn (Ames & Lichter, 2013). In estuaries where alewife were extirpated, inshore gadid populations also disappeared and did not return, suggesting a high predator–prey linkage between gadids and alewife in the northeastern Atlantic and a role of freshwater–marine nutrient subsidies (Table 1; Ames & Lichter, 2013). Further, modeling suggests that if alewife populations increase, their migrations to and from the marine environment could positively impact species of economic importance and conservation concern by acting as a stable food source during unpredictable changes in climate for key marine species (Dias et al., 2019).

### 2.2 Behavior

#### 2.2.1 Migration and foraging

Anadromous alewife range from North Carolina to Newfoundland (Table 1; Collette & Klein‐Macphee, 2002; ASMFC, 2009). YOY alewife typically migrate to marine environments in June and July (Schmidt et al., 1988). Once at sea, juveniles join large intraspecific feeding schools of similar‐sized individuals, with smaller alewives preferring shallower regions compared to larger adults (Table 1; Stone and Jessop, 1981). These feeding schools are mixed‐stock, meaning that several different alewife populations are moving and feeding together (DFO, 1986; Rulifson & Dadswell, 2020). Alewife are also known to form mixed‐species schools with other herrings like menhaden (*Brevoortia* spp.) or blueback herring (*A. aestivalis*; Bigelow & Schroeder, 1953; Rulifson & Dadswell, 2020).

Despite sustaining a critical fishery, there are limited investigations of the marine migratory movement of alewife (Gibson et al., 2017; Huveneers et al., 2016; Neves, 1981; Stone & Jessop, 1992; Tsitrin et al., 2020; Tsitrin et al., 2022; Ogburn et al., 2024). However, a recent acoustic telemetry study demonstrated that alewife from Choptank River, Maryland, migrated northward into Georges Bank and the Gulf of Maine in the summer and moved southward in the fall and winter (Ogburn et al., 2024). These findings validate the yearly migratory patterns inferred from catch and by‐catch data, which suggest that alewife move northward and inshore in the spring, as many prepare to return to fresh water for spawning, and finally offshore and southward in the fall (Table 1; Neves, 1981; Stone & Jessop, 1992). This migration pattern was observed in populations from the Mid‐Atlantic Bight, where summer and fall catches were concentrated in Nantucket Shoals, Georges Bank, and coastal Gulf of Maine regions, suggesting they moved northward from their natal stream (Neves, 1981). Furthermore, winter catches indicated they return to the Mid‐Atlantic coastline in the winter and spring (Table 1; Neves, 1981; Tsitrin et al., 2022). These migratory patterns were further validated by alewife catch data along the coast of Nova Scotia that indicated alewife distribution shifted inshore and northward in spring along the Scotian Shelf and offshore and southward in fall toward the Gulf of Maine and Bay of Fundy (Stone & Jessop, 1992). The extent of offshore overwintering for alewife is still unknown; however, the theorized seasonal distribution of alewife closely resembles American shad migratory movements, which suggests that alewife may use a mixture of inshore and offshore foraging areas annually (Table 1; Neves, 1981).

Alewife movement at sea is presently thought to be regulated by biological factors as they follow zooplankton productivity (DFO, 1986; Neves, 1981; Tsitrin et al., 2022). When alewife feed on plankton, they undertake diel vertical migrations following the vertical movement of zooplankton throughout the water column (Table 1; Neves, 1981; Stone & Daborn, 1987). Stomach content analyses suggest alewife particulate feed on macrozooplankton when water visibility is high during the day and filter feed on microzooplankton during low visibility at night (Table 1; Gilmurray, 1980; Stone & Jessop, 1992).

Recent studies have demonstrated that alewife marine movement is also influenced by water temperature, tidal currents, salinity, and depth (Tsitrin et al., 2022; Turner et al., 2016), suggesting their distributions are constrained by suitable oceanographic conditions. For instance, seasonal spawning migrations are thought to be triggered by water temperatures around 5–10°C (Jessop & Parker, 1988; Tsitrin et al., 2022). Therefore, mature alewife begin spawning migrations toward rivers in late March in the south and progressively later into July further north (Table 1; Cole et al., 1980; Mullen et al., 1986). A recent tagging study also suggests that alewife strongly avoid marine temperatures above 14°C (Tsitrin et al., 2022). In this study, postspawned alewife emigrating from Gaspereau River, Nova Scotia, remained in the Minas basin and foraged in nearshore habitat for 20 days before water temperatures warmed, cueing offshore migration (Table 1; Tsitrin et al., 2022). Similar behavior was observed in American shad, which use cold tidal currents as migration corridors throughout at‐sea movement (Neves & Depres, 1979; Tsitrin et al., 2022).

## 3 BLUEBACK HERRING

### 3.1 Life history

#### 3.1.1 Diet

Blueback herring consume a variety of prey, including pelagic and benthic species; however, their diet is more selective compared to alewife and primarily feed on zooplankton (Mullen et al., 1986; Stone & Daborn, 1987). As juveniles grow and move from their natal rivers into estuaries, larger zooplankton, such as adult copepods (e.g., *Eurytemora affinis* and *Cyclops vernalis*), become the primary diet (Table 1; Mullen et al., 1986). Adults in the marine environment are predominantly planktivorous and continue to consume zooplankton, although they also consume small fishes, eggs of other fish, and crustaceans such as pelagic shrimp (Table 1; Bigelow & Schroeder, 1953; Munroe, 2002).

#### 3.1.2 Growth

Adult blueback herring can reach lengths of 197–322 mm in fork length and weigh 93–468 g for ages 3–12 (Jessop, 1993). Like alewife, adults are sexually dimorphic, where females are slightly larger and have an average total length of 289 mm, whereas males average 277 mm in length (Table 1; Bowlby & Gibson, 2016; Loesch & Lund, 1977). However, it is unclear whether there are further size similarities between alewife and blueback herring. Contradictory reports on size differences in Nova Scotia stated that blueback herring were substantially smaller than alewife on average (Bowlby & Gibson, 2016), whereas a different study found blueback herring to be only marginally smaller (Jessop, 1993). Parameters from the VBGF for blueback herring demonstrate the observed sexual dimorphism and the argument that blueback herring are smaller in size than alewife, with L∞ ranging from 231.33‐ (males) to 259.85 mm (females), K ranging from 0.469 (females) to 0.590 (males), and t_0_ ranging from 0.283 (females) to 0.338 (males; Messieh, 1977).

Most of the growth in blueback herring occurs during the first couple of years at sea and plateaus after they reach sexual maturity. Adults exhibit very little growth between spawning events, such that individuals ranging from 3 to 5 years old only grow an average of 13 mm between annual spawning (Table 1; Mullen et al., 1986; Bowlby & Gibson, 2016). Therefore, blueback herring that have spawned multiple times are smaller for their given age, particularly in weight, suggesting an energetic trade‐off between frequent spawning and at‐sea growth (Table 1; Bowlby & Gibson, 2016). Sex‐specific variation exists within age at first reproduction, where maturation takes 4–5 years for females and 3–4 years for males. For instance, the highest proportion of first‐time male spawners is at ages 3 and 4, accounting for 46.9% and 49.8% of the individuals in each age class, respectively (Marcy, 1969), whereas most first‐time female spawners are older, ages 4 and 5, and account for 74.6% and 16.4% of individuals observed at each age class during spawning events (Marcy, 1969). The causes for this variation remain unclear (Bowlby & Gibson, 2016).

#### 3.1.3 Spawning

Blueback herring are iteroparous and will spawn up to four times before death (Table 1; McBride et al., 2014; Bowlby & Gibson, 2016). They exhibit a degree of spawning site fidelity and will typically return to the same river to spawn (Table 1; Bozeman Jr & Van Den Ayvle, 1989); however, the frequency of return and the proportion that stray to novel spawning sites are unclear. It is speculated that blueback herring rely on olfactory mechanisms, similar to alewife; however, their migratory cues back to natal rivers are not well understood. Additionally, it is unknown whether ovary weight and fork length are related, but research suggests fecundity positively correlates with age and decreases with increasing latitude when measured gravimetrically (Table 1; Jessop, 1993). The length of the spawning season prolongs with increasing latitude and may be a function of cooler northern temperatures (Loesch & Lund, 1977; Lynch et al., 2015), likely compensating for the decreased fecundity at these latitudes.

Although spawning typically occurs in freshwater (Bigelow & Schroeder, 1953), blueback herring will also fertilize eggs in slightly brackish (Kuntz & Radcliffe, 1918) and fully brackish waters (Breder, 1948). Regardless of salinity, studies suggest that spawning occurs beyond the influence of the tide (Bigelow & Schroeder, 1953; Hildebrand, 1963; Hildebrand & Schroeder, 1927). Despite the overwhelming similarities between alewife and blueback herring, there can be some spatial and temporal isolation between the two species during spawning events (Loesch & Lund, 1977). Both species can differ spatially by spawning in different systems or within the same river system but in different areas depending on spawning habitat preferences (Loesch & Lund, 1977). Temporal isolation occurred during the spawning migration to the Tusket River, Nova Scotia; alewife returned to spawn more than 4 weeks earlier than blueback herring (Table 1; Bowlby & Gibson, 2016).

#### 3.1.4 Mortality

In situ observations during field studies in Nova Scotia suggest that the maximum age of blueback herring is variable. In the Tusket River, the oldest individual observed was 8 years old (Bowlby & Gibson, 2016); however, during sampling across the Tusket, Mactaquac, Gaspereau, and Margaree rivers, the maximum age observed was 12 (Table 1; Jessop, 1993). Females tend to live longer than males, and males are more abundant in younger age classes (3–5 years), whereas the abundance of older female blueback herring (7+ years) tends to be far greater (Loesch & Lund, 1977).

As forage fish, blueback herring provide an important connection between marine and estuarine food webs, transferring nutrients from their oceanic zooplankton food source to coastal piscivore predators (Ames & Lichter, 2013). Death in the marine environment is largely due to predation by a variety of predators, including spiny dogfish (*Squalus acanthias*), Atlantic cod (*Gadus morhua*), silver hake (*Merluccius bilinearis*), white hake (*Urophycis tenuis*), Atlantic halibut (*Hippoglossus hippoglossus*), bluefish (*Pomatomus saltatrix*), weakfish (*Cynoscion regalis*), striped bass (*Morone saxatillis*), seals, gulls, and terns (Munroe, 2002).

### 3.2 Behavior

#### 3.2.1 Migration and foraging

Blueback herring have a marine range from Florida (Hildebrand & Schroeder, 1927) to Nova Scotia (Table 1; Bigelow & Schroeder, 1953; Rulifson & Dadswell, 2020), where prespawned individuals spend 2–5 years growing and feeding after leaving their natal rivers (Loesch, 1987). Despite the extensive proportion of their lives spent at sea, relatively little is known about blueback herring marine ecology in comparison to their freshwater, riverine migrations (Bethoney, Stokesbury, & Cadrin, 2014; Neves, 1981; Rulifson & Dadswell, 2020; Stone & Jessop, 1992). However, recordings of long‐distance feeding migrations show migratory routes from North Carolina to Nova Scotia, which align with the routes of American shad (Rulifson & Dadswell, 2020). There is also evidence of the separation into migratory contingents, where a proportion of blueback herring are semi‐resident in estuarine regions near their natal rivers and use inshore embayments for feeding instead of undertaking large offshore migrations (Table 1; Stone & Jessop, 1992; Rulifson & Dadswell, 2020). Due to the complex movement dynamics in each population, summer foraging aggregations consist of mixed stocks and therefore include multiple contingents from different spawning rivers (Table 1; Rulifson & Dadswell, 2020).

When foraging in the marine environment, blueback herring benefit from schooling behavior (i.e., optimizing feeding and avoiding predators, etc.) and group into single‐species aggregations at sea or school with alewife (Table 1; Bethoney et al., 2013; Rulifson & Dadswell, 2020). During summer feeding migrations, blueback herring follow zooplankton throughout the marine environment. There is evidence that blueback herring undertake diel vertical migrations (Jessop, 1990), which mimic the diel movements of zooplankton (Table 1; Neves, 1981). However, the availability of suitable water temperatures may limit their vertical feeding migrations (Stone & Jessop, 1992). Similar to their vertical movements, the horizontal migrations of blueback herring are likely tied to zooplankton concentrations at different latitudes throughout the year, outside of the spawning migration (Neves, 1981; Stone & Jessop, 1992). Researchers in the Minas basin in Nova Scotia have found evidence of filter‐feeding among anadromous blueback herring, although they may also undertake active foraging in the marine environment (Table 1; Stone & Daborn, 1987).

After foraging at sea during summer, blueback herring move southward in the fall and aggregate in offshore overwintering sites in the Middle Atlantic Bight (Neves, 1981) and along the Scotian Shelf from the Gulf of Maine to the Bay of Fundy (Stone & Jessop, 1992). Neves (1981) speculated that temperature and availability of zooplankton drive the selection of specific overwintering sites. Although blueback herring are typically associated with inshore habitats and remain in shallower waters than alewife (Bethoney, Stokesbury, Schondelmeier, et al., 2014; Neves, 1981), during winter, they are found comparatively deeper, likely seeking warmer bottom water, preferring water >5°C offshore (Stone & Jessop, 1992). This temperature preference suggests that blueblack migration strongly follows temperature contours in the northern limit of their range (Canada; Table 1; Stone & Jessop, 1992).

In early spring, blueback herring start to occupy mid‐depth, nearshore waters along the Atlantic coast as they move northward to reach spawning rivers in late spring (Neves, 1981; Stone & Jessop, 1992). The timing of arrival for the spring spawning migration is, again, thought to be temperature‐driven, timing their movements to coincide with an optimal water temperature range of 14–22°C for spawning (Table 1; Bi et al., 2021, Loesch & Lund, 1977, Ogburn et al., 2024). Blueback herring prefer warmer spawning temperatures than alewife, and so, they generally arrive later than alewife in their shared rivers (Bigelow & Schroeder, 1953). For blueback herring, this leads to a late‐April arrival in spawning rivers (although this varies), and they can remain at these sites for several months into the summer before they return to sea (Jones et al., 1978).

## 4 AMERICAN SHAD

### 4.1 Life history

#### 4.1.1 Diet

On their migration back to sea, adult American shad prey on smaller freshwater fish, including shield darters (*Percina peltata*) and, at times, cannibalize juvenile American shad (Table 1; Chittenden Jr., 1976b). Beyond these nearshore data, there are only a few studies to rely on for characterizing American shad diet in the marine environment; however, adults are known to return to their planktonic diet at sea and primarily feed on copepods and mysids (Walburg & Nichols, 1967). Stomach content analyses have also shown that adults prey on small fish (Table 1;  Facey & Van Den Avyle, 1986; Walburg & Nichols, 1967). More information is needed to better understand American shad diet at sea during juvenile and adult life stages.

#### 4.1.2 Growth

In addition to freshwater environments, YOY American shad also use estuarine areas as nursery grounds (Crecco et al., 1983; McCormick et al., 1996), typically in low‐velocity waters where food availability is higher (Limburg, 1996). Juveniles grow to 38–114 mm long before they emigrate into the marine environment in autumn (Bigelow & Schroeder, 1953; Mitchell, 1925), suggesting that growth and factors associated with size influence the timing of seaward emigration (Chittenden Jr., 1969; Miller et al., 1973). Most growth occurs at sea, where prespawned fish spend 2–6 years until they reach sexual maturity (Table 1; Bigelow & Schroeder, 1953; Walburg & Nichols, 1967). Males reach maturity younger than females, at around 2 years or when they reach 290 mm (on average), whereas females mature in their third or fourth year when they are closer to 400 mm (Walburg & Nichols, 1967). As such, American shad are sexually dimorphic; females are typically larger than males within their age class (Du et al., 2023). Compared to alewife and blueback herring, American shad are the largest among these Alosidae, and adults can grow up to 760 mm and weigh up to 5500 g (Bigelow & Schroeder, 1953). Their maximum size depends on latitude, and northern populations reach larger sizes than their southern contingents (Table 1; Gilligan‐Lunda et al., 2021; Poulet et al., 2023; Robins et al., 1986). The VBGF parameters for American shad reinforce their size difference when compared to the parameters for alewife and blueback herring, with an average of 481 mm for L∞, 0.44 for K, and −0.32 for t_0_ (Gilligan‐Lunda et al., 2021).

#### 4.1.3 Spawning

American shad exhibit spawning site fidelity to their natal rivers (Table 1; Carscadden & Leggett, 1975; Hill, 1959; Nichols, 1966; Talbot, 1954), with low levels of straying (3%; Melvin et al., 1986) in some populations (Mansueti & Kolb, 1953; Williams & Daborn, 1984). Spawning strategies also differ latitudinally where northern populations are iteroparous and can spawn five (Grote et al., 2014; McBride et al., 2016) to seven times (Provost, 1987), whereas southern populations are semelparous (reproduce once then die; Table 1; Leggett & Carscadden, 1978; Poulet et al., 2023). Semelparous populations include those that spawn in the St. Johns River, Florida (Limburg et al., 2003) and the Ogeechee River, Georgia (Sykes, 1956). This semelparity observed in southern populations appears to be an adaptation to the consistent weather in the south, resulting in a higher probability of successful recruitment and consequently higher fecundity, when defined as the rate of successful recruitment (Carscadden & Leggett, 1975; Roff, 1992).

#### 4.1.4 Mortality

American shad have been reported to live up to 13 years, and there is no recorded evidence of sexual dimorphism regarding maximum age, like in blueback herring populations (Altman & Dittmer, 1962). However, the natural mortality rate does differ based on latitude, where northern populations reach an older maximum age due to alternate reproductive styles, much like alewife (Gilligan‐Lunda et al., 2021; Poulet et al., 2023). Other barriers that limit survival include predation, target fisheries, and by‐catch (Table 1; Bailey et al., 2004; Bethoney et al., 2013). At sea, American shad are preyed on by sharks, bluefin tuna (*Thunnus thynnus*), kingfish (*Scomberomorus cavalla*), and porpoises in the southern United States (Walburg & Nichols, 1967). Passive tagging studies on American shad have also revealed that dogfish (*S. acanthias*) and Atlantic cod (*G. morhua*) also prey on American shad (Dadswell, 1990). Additionally, there are records of seals preying on American shad as they begin their spawning runs into river mouths (Dadswell, 1990). Although northern American shad are iteroparous, like gaspereau, it is not uncommon for adults to die on their spawning migration. For instance, in northern New Jersey, dead, egg‐bound females were found during their migration upstream to spawning grounds, likely attributable to starvation (Chittenden Jr., 1976a). Therefore, attrition of American shad when they enter freshwater forms a key part of the species' marine demography.

### 4.2 Behavior

#### 4.2.1 Migration and foraging

American shad exhibit a complex marine migration pattern influenced by both environmental cues and intrinsic factors. They undertake extensive seasonal migrations in surface waters (Neves & Depres, 1979) along the northwest Atlantic, spanning from Newfoundland and Labrador (Dempson et al., 1983; Limburg et al., 2003) to Florida (Table 1; Williams & Bruger, 1972). YOY juveniles either spend the first year in the lower estuary of their natal river (Hoffman et al., 2008; Leggett & Whitney, 1972) or exhibit short residency, staying within fresh water for the summer, then migrating to sea in the fall (Greene et al., 2009; Neves & Depres, 1979). Upon entering the marine environment, juveniles join large intraspecific schools of immature and postspawning adults for feeding and growth (Bigelow & Schroeder, 1953; Dadswell et al., 1987). As water temperatures cool later into the fall, schools migrate in surface waters (Bigelow & Schroeder, 1953) southward to overwintering sites, which include deeper waters, 40–175 km offshore Florida, the Mid‐Atlantic Bight, and along the Scotian Shelf (Collette & Klein‐Macphee, 2002; Dadswell et al., 1987). The aggregations in these overwintering sites are heterogeneous mixtures of American shad populations from many rivers (Dadswell et al., 1987).

Spawning can occur when water temperatures cool to 8–26°C (Walburg & Nichols, 1967); however, peak spawning begins when water temperatures fall between 12 and 21°C (Jessop, 1975; Jones et al., 1978). Therefore, spawning migrations are temporally influenced, and southern populations begin migrating toward natal rivers in January, with some reaching the southern limit of their range in the St. Johns River, Florida (Table 1; Limburg et al., 2003). Northern populations begin migrations to spawning rivers progressively later into the spring as latitude increases (Limburg et al., 2003). During the summer, spawning continues upstream from the Delaware River to the St. Lawrence River, and northern American shad populations concentrate in the surface waters of foraging grounds (Leim, 1924; Themelis, 1986) in the inner Bay of Fundy, the inner Gulf of St. Lawrence, and off of Newfoundland and Labrador (Table 1; Dadswell et al., 1987).

Foraging behavior varies based on life stage and habitat. Walter III and Olney (2003) demonstrated that adult stomach fullness index was highest in the marine environment (Table 1), followed by estuarine habitats, then freshwater, indicating the majority of feeding takes place at sea. There is little information about juvenile stomach fullness; however, juveniles are known to form feeding schools along the coast (Greene et al., 2009; Neves & Depres, 1979) and demonstrate diel feeding patterns primarily foraging in the evening (Johnson & Dropkin, 1996; Massmann, 1963). Additionally, planktivorous adults feed both passively by filtering prey as they swim and by actively ambushing (Harris & McBride, 2009), suggesting that American shad mainly consume what they encounter and feed if suitable prey is available (Atkinson, 1951; Leim, 1924; Walter III & Olney, 2003).

## 5 THREATS AND FISHERIES MANAGEMENT

### 5.1 Threats

**FIGURE 2:**
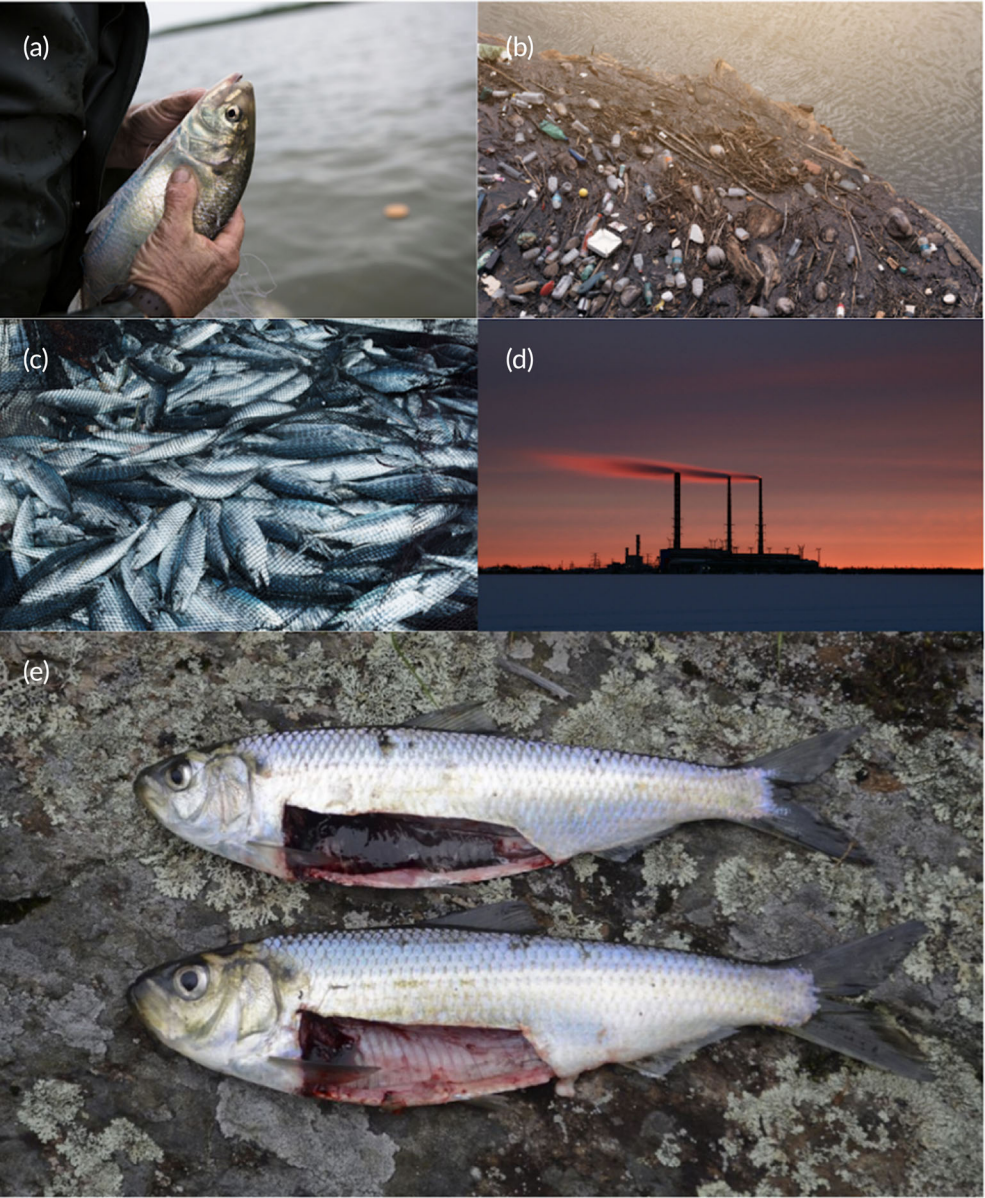
Threats facing diadromous Alosids, including alewife, blueback herring, and American shad in marine environments. (a) commercial fisheries, (b) habitat degradation, (c) by‐catch in Atlantic mackerel and Atlantic herring commercial fisheries, (d) global climate change, and (e) misidentification and hybridization of alewife and blueback herring (collectively known as gaspereau and river herring; photo credit: [a]–[d] Canva, [e] Christopher Bartlett).

The IUCN most recently assessed alewife and American shad as “least concern,” whereas blueback herring were classified as “vulnerable” (Table 2). Despite mounting concern surrounding population declines, federal protection under the Species at Risk Act (SARA) in Canada and the Endangered Species Act in the United States have not yet occurred for these species due to data deficiencies (Table 2). Overfishing was historically the primary contributor to population declines of alewife, blueback herring, and American shad (Figure 2a; Bailey et al., 2004). Specifically, the commercial freshwater fisheries of gaspereau in Canada and the United States heavily contributed to population declines in recent history (DFO, 2021). Further, the estimated exploitation rates for Canadian alewife and blueback herring in the late 1990s were as high as 89% based on catch, escapement, and run size estimates for each year (DFO, 2021). Additional information regarding the effects and management of Alosid fisheries can be found in the comprehensive review by Hare et al. (2021). The decimation caused by freshwater fisheries should be viewed as a cautionary tale for marine fisheries to avoid similar population declines. Despite the continued exploitation of Alosids at the commercial scale, more recently, the most predominant threats to these species include habitat degradation (Figure 2b), by‐catch in commercial fisheries (Figure 2c), and climate change impacts (Figure 2d; ASMFC, 2012; Belding & Corwin, 1921; Bethoney et al., 2013; Hare et al., 2021; Limburg & Waldman, 2009; Spares et al., 2023).

**TABLE 2:** Status and commercial fishery summary table for alewife (*Alosa pseudoharengus*), blueback herring (*Alosa aestivalis*), and American shad (*Alosa sapidissima*) in Canada and the USA.

Jurisdiction	Alewife	Blueback herring	American Shad
IUCN status	Least concern	Vulnerable	Least concern
Canada			
SARA status	Not listed	Not listed	Not listed
Commercial fisheries	Yes, the majority occur in fresh water with only a few small‐scale marine fisheries Permitted gear: gillnet, dip‐net, square net, trap net, and weir		
Recreational fisheries	Yes, managed federally Regulations regarding location, gear (permit dip‐net, gillnet, square net, trap net, or weir), and seasonal closures Recreational harvests permitted		
By‐catch^a^	N/A	N/A	N/A
United States			
ESA status	Not listed	Not listed	Not listed
Commercial fisheries	No, nearly all of the eastern states closed their commercial fisheries in 2012, and state‐level moratoria are now in place Permitted gear for the few that remain: small‐mesh bottom trawlers and larger‐scale paired mid‐water/ bottom trawlers		
Recreational fisheries	Yes, managed by state Regulations for location, and gear (permit dip‐nets, hook‐and‐line) Majority do not permit recreational harvests		
By‐catch^b^	2.8 million lb	2.8 million lb	10,000 lb

Abbreviations: ESA, Endangered Species Act; SARA, Species at Risk Act.

^a^ By‐catch landings for the Atlantic mackerel and Atlantic herring commercial fisheries in Canada are not openly available.

^b^ All estimated by‐catch landings were from the 2022 by‐catch reports of Atlantic herring and Atlantic mackerel commercial fisheries and published in 2023 by the Atlantic States Marine Fisheries Commission (ASMFC).

Habitat degradation results from several anthropogenic activities, such as ditching in coastal areas and other shoreline modifications, like filling/stabilizing and coastal hardening (German et al., 2023). Coastal ditching is particularly pervasive to gaspereau and American shad in marine systems due to its contributions to increased pollution, changes in hydrology, and the loss of habitat complexity (Frankensteen, 1976; NCDMF, 2022). By‐catch, however, is the primary threat in the marine environment, and all three species are frequently caught in the North American Atlantic mackerel (*Scomber scombrus*) and Atlantic herring (*Clupea harengus*) fisheries (NOAA Fisheries, 2023). Alewife from Chesapeake Bay migrate through several fisheries jurisdictions during their postspawning migration to the Bay of Fundy and Gulf of Maine (Ogburn et al., 2024). Trawl surveys from the Gulf of Maine confirm a substantial number of incidental catches of alewife overlap with their migratory temporal distribution (Bethoney, Stokesbury, & Cadrin, 2014). Additionally, in 2022, an estimated 2.8 million pounds of gaspereau and 110,000 pounds of American shad were landed as by‐catch in the United States (ASMFC, 2023).

Climate change is also a growing concern for Alosids in the northwest Atlantic (Gilligan‐Lunda et al., 2021), impacting marine migration and behavior and contributing to habitat degradation. Warming temperatures, among many other cascading effects of climate change, are predicted to reduce areas of suitable habitat across the North American Atlantic coast, particularly for spawning habitat (Lynch et al., 2015; McHenry et al., 2019). In the marine environment, warming surface water temperatures influence the distribution and abundance of zooplankton communities, negatively impacting the foraging behavior of Alosids (Barton et al., 2016; Beaugrand et al., 2015; Beaugrand & Ibanez, 2004; Poloczanska et al., 2013; Richardson & Schoeman, 2004). Specifically, areas along the primary migratory routes of gaspereau are rapidly warming (Hinson et al., 2022; Pershing et al., 2015) and changes in ocean currents (IPCC, 2021) that can affect gaspereau energy budgets (Cobb, 2020; Hare et al., 2016), and likely driving shifts in predator–prey relationships at sea (Coutant, 1990; Nack et al., 2019; Portner & Peck, 2010).

### 5.2 Management of commercial fisheries

Commercial fishing of gaspereau is federally permitted in Canada and primarily occurs inland in rivers and tidal waters of New Brunswick and Nova Scotia. However, several small marine fisheries operate on the Atlantic coast of Nova Scotia along the eastern shore (DFO, 2021). The management of these fisheries in Canadian waters has remained the same since 2001 despite federal landing reports from the Gulf region (1917–2019) showing a decline (DFO, 2022). Due to insufficient data, it is unknown whether the decline accurately reflects abundance (DFO, 2022). Further, for most stocks in the Maritimes Region, commercial landing records are the only available source of information (Gibson et al., 2017). Likewise, the major commercial fisheries for American shad were historically located in large spawning rivers on the east coast of Canada (Saint John, St. Lawrence) and the embayments of the upper Bay of Fundy (Dadswell, 1990). Currently, the remaining commercial American shad fisheries in Canada are restricted to freshwater environments and operate on a small scale, both in terms of size and landings (Chaput & Bradford, 2003).

In 2012, numerous states along the eastern coast of the United States closed their commercial fisheries for alewife and blueback herring to address the decline in their populations (Maryland, 2024). Today, most states have implemented state‐level moratoria on the commercial fishing of these species (NMFS, 2019). In 2014, catch limits for gaspereau and American shad were created for other commercial fisheries harvesting these species as by‐catch. In the Atlantic mackerel (*S. scombrus*) fishery, the combined catch limit for gaspereau and American shad is set at 89 metric tons (mt). However, if the total mackerel landings exceed 10,000 mt, this cap increases to 155 mt (NOAA Fisheries, 2023). If 95% of the cap is harvested, a 20,000‐lb mackerel possession limit will become effective for the remainder of the fishing year (NOAA, 2023). In the Atlantic herring (*C. harengus*) fishery, the collective catch limit for gaspereau and American shad is area‐ and gear‐specific. There is a midwater trawl cap of 86 mt for the Gulf of Maine, 13 mt for Cape Cod, 124 mt for Southern New England, and a bottom trawl cap of 89 mt for Southern New England (NOAA, 2023). If 95% of the cap is harvested in any area, a 2000‐lb Atlantic herring possession limit for that area and gear will become effective for the remainder of the fishing year (January 1–December 31).

Contrasting the two management regimes suggests that the United States is taking a more cautious approach to protect these species in marine regions. Although movements between the United States and Canada have been documented, the regulations are vastly different across borders. This contrast between countries suggests much room for improvement regarding the assessment and management of Alosids in Canadian marine waters.

### 5.3 Management of recreational fisheries

In Canada, recreational fisheries for gaspereau and American shad occur in freshwater systems and are managed federally. There are regulations regarding location, gear (dip‐net, gillnet, square net, trap net, or weir), and seasonal closures (Table 2; NMFS, 2019; Government of Canada). Daily catch limits are often in effect despite the lack of licensing or reporting requirements and recreational harvests are not well documented (Table 2; NMFS, 2019). In the United States, recreational fisheries (most commonly using dip‐nets and hook‐and‐line) vary between states and are reported individually (NMFS, 2019). Most states do not permit the recreational harvest of alewife or blueback herring (Table 2; NMFS, 2019). Some states (Maine, New Hampshire, Rhode Island, New York, and South Carolina) are, however, exempt from the Atlantic States Marine Fisheries Commission (ASMFC) requirements, and small recreational harvests are permitted through a sustainable fisheries management plan (NMFS, 2019). These states were exempt because their catches are commonly used as bait for the recreational striped bass fishery and as food (NMFS, 2019). There are few records documenting recreational catches and management of American shad in the United States (ASMFC, 2023).

Information pertaining to marine recreational fisheries in both countries is virtually nonexistent. Assessments are required to determine the extent of marine recreational fisheries, and similar regulations should be developed and implemented to ensure that all forms of recreational fishing do not put additional strain on Alosid populations.

## 6 KNOWLEDGE GAPS AND RESEARCH NEEDS

Here, we identify knowledge gaps in the marine ecology of Alosids that are potentially hindering effective management. Gaps include a thorough understanding of marine movement, the effects of climate change on Alosid behavior and migration, population dynamics (i.e., genetic diversity, stock mixing), and the grouping of alewife and blueback herring in management efforts (Table 3). We highlight research needs that will address these gaps and contribute to developing comprehensive conservation strategies and sustainable management practices for Alosid populations.

**TABLE 3:** Summary of identified paucities regarding the marine ecology of anadromous alewife (*Alosa pseudoharengus*), blueback herring (*Alosa aestivalis*), and American shad (*Alosa sapidissima*).

Knowledge gap		Description	Reference
Marine movement		Little is understood about marine distribution, habitat preferences, thermal boundaries, and precise migration routes (seasonal patterns) Extent of alewife overwintering is unknown The cues that influence movement (lunar, environmental, or physical) are understudied	ASMFC, 2012; Neves, 1981
Effects of climate change	Behavior	Unknown response to changes in food distribution and prey availability Changing ocean currents and rising water temperatures have unknown consequences on habitat use Few assessments on resilience to warming oceans and population‐specific eurythermality	Bi et al., 2021; Bindoff et al., 2022; Behrenfeld & Falkowski, 1997; Jessop, 1975; Jones et al., 1978; Loesch & Lund, 1977; Richardson & Schoeman, 2004; Tsitrin et al., 2022
	Migration	Unknown effects of sea surface temperature changes on spatial distribution and migration patterns Outdated research on temperature‐driven movement	Jessop, 1975; Loesch & Lund, 1977; Jones et al., 1978; Ogburn et al., 2024; Bi et al., 2021; Tsitrin et al., 2022
Population dynamics	Genetic diversity	Genetic integrity and diversity within individual stocks are unknown Are populations from different rivers genetically distinct?	Dadswell et al., 1987; Bethoney et al., 2013; Hasselman & Limburg, 2012; Rulifson & Dadswell, 2020; Bozeman Jr & Van Den Ayvle, 1989
	Stock mixing	Degree of straying from natal rivers is unknown for blueback Stock mixing into heterogeneous populations or single‐species aggregations during marine migrations is unclear	(Bethoney et al., 2013; Dadswell et al., 1987; Rulifson & Dadswell, 2020).
Distinguishing alewife from blueback and hybridization		Alewife and blueback are managed as one species due to morphological similarities and spatiotemporal overlap Inaccurate stock assessments and misinterpretation of population trends due to misidentification	Bigelow & Schroeder, 1953; Messieh, 1977; Collette & Klein‐Macphee, 2002; Lynch et al., 2015; Hare et al., 2021

### 6.1 Marine movement

In Atlantic Canada, these migratory clupeiformes exit rivers to the Bay of Fundy, Scotian Shelf, Gulf of St. Lawrence, and the Labrador Sea. Despite spending a significant proportion of their lives in the ocean, little is known about their marine distribution, thermal boundaries, habitat preferences, and precise migration routes (ASMFC, 2012). Understanding the factors that drive the marine distribution of migratory Alosids is necessary for predicting marine movement and determining the vulnerability to various stressors. For many species, including salmonids (Furey et al., 2015; Kocik et al., 2009b; Rikardsen et al., 2021), Atlantic cod (Staveley et al., 2019), European eel (Righton et al., 2016), bluefin tuna (Lutcavage et al., 2000), sharks (Espinoza et al., 2015; Lara‐Lizardi et al., 2020; Weng et al., 2007), and Atlantic sturgeon (Kessel et al., 2017; Rothermel et al., 2020), passive and active tagging has been key to providing insights into where and when animals are occurring in coastal and marine waters. Such techniques would be valuable for better understanding the marine migration patterns of Alosids as well. Few tracking studies on alewife and American shad have been conducted; however, these have mostly been confined to freshwater or coastal zones or did not report on marine detections (Bunch et al., 2023; Gahagan & Bailey, 2020; Mack et al., 2020; McCartin et al., 2019; Tsitrin et al., 2022). Further, satellite tagging is intractable for Alosids, and these species are moderately sensitive to handling; therefore, they require more refined protocols for tagging research (Tsitrin et al., 2020).

However, in a recent study, Ogburn et al. (2024) acoustically tagged 50 alewives in Chesapeake Bay to monitor their marine migration to the Gulf of Maine. The study revealed that this subset of alewife traversed areas subject to significant fishing pressures (incidental by‐catch in trawls), as well as regions experiencing rising sea surface temperatures. The authors advocate for the continued use of large‐scale acoustic telemetry networks in the northwest Atlantic to gather marine data on blueback herring and American shad, which is essential for addressing the identified threats and data gaps. Moreover, the infrastructure for acoustic telemetry has recently expanded, and several broad‐scale arrays and opportunities for finer‐scale tracking exist in the northwest Atlantic (Bangley et al., 2020). Telemetry alone can provide valuable insights into marine movement, but when combined with other data sources and technologies, it becomes a more powerful, better‐informed, and collaborative approach to answering unresolved questions (Bangley et al., 2020). By coupling telemetry studies with historical fisheries catch data, oceanographic data, and other methods such as otolith geochemical signatures, researchers could better understand the spatial marine ecology of Alosids. The knowledge gained would help inform fisheries management and conservation efforts, such as habitat restoration, by‐catch mitigation, and sustainable harvest practices across their range.

### 6.2 Climate change

Warming sea surface temperatures increase the urgency of investigating Alosid migration corridors and patterns. Temperature may significantly affect marine movement and habitat use, especially if thermal barriers constrain key areas and usable habitat becomes restricted. Given the wide latitudinal ranges of these Alosid species, a greater understanding of population‐specific eurythermality would be valuable to understanding their tolerance to different thermal regimes along the Atlantic coast. In addition to raising sea surface temperature, climate change impacts ocean currents, and in turn, prey availability, by altering the distribution of phytoplankton and zooplankton communities (Behrenfeld & Falkowski, 1997; Bindoff et al., 2022; Richardson & Schoeman, 2004). North Atlantic right whales, a megafaunal filter feeder, have shifted their foraging range from the Bay of Fundy to the Gulf of St. Lawrence in recent years (Davies & Brillant, 2019), illustrating how planktivores may have to rapidly adapt to the impacts of climate change. The limited studies on temperature‐driven Alosid movement are focused on freshwater or coastal movement; therefore, further research is needed to understand how increasing marine temperatures impact Alosid migration timing and route selection, either directly through thermal barriers or indirectly through prey distribution. (Bi et al., 2021; Jessop, 1975; Jones et al., 1978; Loesch & Lund, 1977; Tsitrin et al., 2022).

Similar to determining precise migration routes using acoustic telemetry, integrating oceanographic data into spatial analysis, such as sea surface temperatures, ocean currents, and planktonic community abundance, would help researchers better understand the environmental impact on Alosid movement. Additionally, laboratory‐based experimental studies on adult Alosids could help refine our knowledge of their thermal tolerances by exploring temperature preferences, performance in varying temperatures, and critical thermal maximums. Upper thermal tolerances of larval and juvenile American shad have been explored in laboratory settings (Bayse et al., 2019; Leach & Houde, 2005); however, this again highlights the need for research on the marine phase of Alosid life history. Assessing the thermal preferences and adaptability of adult Alosids through laboratory experiments, and combining this with oceanographic data, would enhance our understanding of Alosid marine habitat use.

### 6.3 Population dynamics

Further research is needed on the genetic diversity and stock mixing of Alosids, as offshore aggregations can consist of single species or heterogeneous mixtures of populations during foraging (Bethoney et al., 2013; Dadswell et al., 1987; Rulifson & Dadswell, 2020). For example, stock mixing remains unclear for blueback herring, as some populations make large marine migrations, whereas others remain near their natal streams in estuaries—creating mixed stocks from different natal rivers (Bethoney et al., 2013; Rulifson & Dadswell, 2020). Additionally, some schools mix with schools of alewife or other herring (Bigelow & Schroeder, 1953; Rulifson & Dadswell, 2020). A greater resolution of genetically distinct populations of alewife, blueback herring, and American shad is required to determine their abundance in these mixed stocks (Hasselman & Limburg, 2012). Understanding the genetic diversity and stock mixing of Alosids is essential for determining the genetic integrity of different populations and how they may respond to environmental changes, such as climate change, and pressures from commercial fisheries. Recent studies focused on the genetic stock composition of alewife and blueback herring caught as by‐catch in commercial fisheries demonstrated high by‐catch mortality rates (from 2012 to 2015 4.6 and 1.2 million, respectively) and serve as an excellent example of the usefulness of genetics‐based studies (Hasselman et al., 2015; Reid et al., 2023). These data would help identify management units and interactions between populations and species, inform the genetics of potential supplementation programmes, and reveal the percentage of populations within different stocks, which is essential for effective fisheries management. Several approaches to fulfilling this research could be range‐wide population genetic sampling to assess diversity and gene flow; hybridization studies focused on assessing the extent of hybridization between alewife and blueback herring; and field collections of fin clips, scales, or tissue sampling during fisheries surveys to conduct mixed‐stock analysis (e.g., Brown et al., 1999; Hasselman et al., 2014; Kan et al., 2017; McBride et al., 2014; Palkovacs et al., 2014).

### 6.4 Grouping of alewife and blueback herring

Alewife and blueback herring are often grouped and managed together, termed “river herring” or “gaspereau,” due to their morphological similarities (Figure 2e) and spatiotemporal overlap (Hare et al., 2021; Lynch et al., 2015). Despite the thorough description of the morphological differences between alewife and blueback herring (Bigelow & Schroeder, 1953), researchers, harvesters, and management lack the tools needed to effectively differentiate these species without conducting a time‐consuming genetic analysis or killing the fish by examining the peritoneal lining (Bigelow & Schroeder, 1953; Messieh, 1977). Separating alewife and blueback herring is crucial for accurately assessing their population dynamics, habitat preferences, and response to environmental change. By failing to distinguish alewife from blueback herring, management strategies may overlook species‐specific vulnerabilities and conservation opportunities. Further, misidentification can lead to misinterpretation of population trends and ineffective identification of hybridization occurrences. To resolve the issues with grouping alewife and blueback herring, each should be managed separately, integrating ecological and behavioral data along with their spatiotemporal distributions and ecological interactions. Additionally, machine learning algorithms could aid in discerning these cryptic species and refine identification in datasets (e.g., Burroughs et al., 2024; Johnson et al., 2017; Marrable et al., 2022).

## 7 CONCLUSION

Within the Atlantic, Alosids have historically been an overlooked species, and although most of their life is spent at sea, there are key knowledge gaps regarding their marine ecology. Here, we have synthesized what little is known about the time spent in marine ecosystems for alewife, blueback herring, and American shad, including their diet, growth, mortality, migrations to spawning sites, and foraging behaviour. We also identified several threats these species face, such as by‐catch, global climate change, and habitat alteration, and highlighted the need to better understand these impacts to generate mitigation strategies. Additionally, we described the key knowledge gaps that are hindering effective management of Alosids during the marine phase of their life. If these species continue to be understudied, Alosids in the Atlantic could experience an invisible collapse, which could go unnoticed by fisheries managers and the public. Addressing these threats and knowledge gaps through targeted research on marine ecology and movement patterns is essential for the development of informed management strategies aimed at conserving and restoring Alosid populations.

## AUTHOR CONTRIBUTIONS

R.J.L., O.D.P.N.‐G., M.L.P., and J.B.R. contributed to the development of the paper topic. C.R.B. drafted the abstract, contributed to all paper sections, wrote Shad behavior, prepared Tables 1 and 3, and prepared the manuscript for submission. A.J.A.S. wrote the American shad life history section, the threats and management section, prepared Table 2, and reviewed all sections. C.S.B contributed to blueback behavior, threats and management, and edited all sections. N.K. handled the knowledge gaps and future research section. J.B.R. addressed alewife behavior and edited all sections. P.M.B.M. contributed the American shad life history section. O.D.P.N.‐G. authored the alewife life history section and verified references. M.L.P. and R.J.L. wrote the introduction and edited all sections.
